# Ultrasound-responsive hydrogel microcarriers co-loading dexamethasone and urate oxidase for localized gout management

**DOI:** 10.3389/fchem.2026.1804709

**Published:** 2026-03-25

**Authors:** Weijing Zhang, Wei Liao, Shuangxiu Tan, Di Zhao, Jing Yao, Weiyu Chen, Jing Zhang, Yuanyuan Xie

**Affiliations:** 1 Department of Ultrasound Medicine, Nanjing Drum Tower Hospital, Affiliated Hospital of Medical School, Nanjing University, Nanjing, China; 2 Children’s Hospital of Nanjing Medical University, Nanjing, China; 3 College of Mechanical and Electronic Engineering, Nanjing Forestry University, Nanjing, China; 4 State Key Laboratory of Pharmaceutical Biotechnology, Division of Sports Medicine and Adult Reconstructive Surgery, Department of Orthopedic Surgery, Drum Tower Hospital affiliated to Medical School of Nanjing University, Nanjing, China; 5 Clinical Stem Cell Center, Nanjing Drum Tower Hospital, Affiliated Hospital of Medical School, Nanjing University, Nanjing, China

**Keywords:** bone repair, cartilage injury, dexamethasone, gout, hydrogel, ultrasound, urate oxidase

## Abstract

**Introduction:**

Gouty arthritis is characterized by the deposition of monosodium urate crystals, which drive not only joint inflammation but also progressive bone erosion and structural damage. Existing therapeutic strategies remain limited by poor local bioavailability and inadequate protection of bone tissue. Hydrogel drug delivery systems offer significant potential for localized gout therapy. However, the co-delivery of anti-inflammatory and urate-lowering agents using hydrogel platforms remains largely unexplored.

**Methods:**

In this study, we propose a novel ultrasound-responsive hydrogel microcarrier (DXM/UOX@MPs) fabricated via microfluidic electrospray, composed of a sodium alginate/N-isopropylacrylamide (NIPAM) double network and co-loaded with dexamethasone (DXM) and urate oxidase (UOX). The microcarriers were designed to be administered intra-articularly and evaluated in gouty rat models. Mechanistically, DXM is slowly released for long-term anti-inflammation, while high-frequency ultrasound triggers NIPAM contraction to release UOX for targeted uric acid degradation.

**Results:**

In gouty rat models, combined therapy with DXM/UOX@MPs and ultrasound achieved superior efficacy. We observed a significant reduction in joint swelling and inflammation in the affected joints. Furthermore, combining the treatment with the inherent cartilage-protective properties of the hydrogel matrix offered a strong protective effect that successfully safeguarded both cartilage and bone from damage.

**Discussion:**

This synergistic strategy addresses key clinical drawbacks, such as poor local bioavailability and inadequate bone tissue protection. By effectively combining sustained anti-inflammation and ultrasound-triggered uric acid degradation, it provides a promising therapeutic approach for gout with high clinical application value.

## 1 Introduction

Gout is a prevalent metabolic arthritis triggered by the deposition of monosodium urate (MSU) crystals in joints and periarticular tissues, which has become a growing global health burden due to the rising incidence of obesity, high-purine dietary patterns (Zhang et al., 2025). The pathological hallmark of gout lies in recurrent acute inflammatory flares, where MSU crystals activate the innate immune response, inducing neutrophil infiltration and the secretion of pro-inflammatory cytokines such as interleukin-1β, ultimately leading to severe joint redness, swelling, and pain (Dalbeth et al., 2021). Clinically, gout management primarily encompasses two core strategies: anti-inflammatory therapy for acute flares and urate-lowering therapy for the chronic phase (FitzGerald, 2025). For acute gouty arthritis, first-line agents include non-steroidal anti-inflammatory drugs (NSAIDs), colchicine, and glucocorticoid to rapidly alleviate inflammation and pain. In the chronic phase, urate-lowering medications such as xanthine oxidase inhibitors and urate oxidase (UOX) are commonly prescribed to reduce serum uric acid (SUA) levels, thereby preventing the formation and further deposition of MSU crystals.

Despite the availability of multiple therapeutic options, current clinical treatments for gout still suffer from notable limitations. In the acute phase, NSAIDs and colchicine often cause severe gastrointestinal adverse effects, and their efficacy is compromised in patients with poor drug tolerance or impaired liver and kidney function. Systemic administration of glucocorticoids may lead to systemic complications such as hyperglycemia, hypertension, and immunosuppression (Li et al., 2020). Among these, Dexamethasone (DXM) is a potent synthetic glucocorticoid widely used across various types of disorders including ulcerative colitis, allergic reactions, and arthritis, due to its anti-inflammatory and immunosuppressive properties. Furthermore, it enables local administration, effectively circumventing the systemic adverse effects. (Rezvanfar et al., 2025). For chronic urate-lowering therapy, long-term medication is required, and some patients may develop drug resistance or fail to achieve the target SUA level (Johnson et al., 2024). Current therapeutic strategies for gout prioritize anti-inflammatory treatment, followed by the initiation of urate-lowering therapy once inflammation has subsided (Dalbeth et al., 2021). In recent years, enzymatic therapy has emerged as an effective therapeutic strategy for patients with gout (Botson et al., 2023; Fan et al., 2023). UOX catalyzes the oxidation of urate into water-soluble allantoin, which is then excreted via the kidneys; this enzyme is characterized by high specificity and high catalytic efficiency. Pegloticase, a PEGylated recombinant mammalian uricase approved in the United States, is capable of sustainably lowering serum uric acid levels and rapidly dissolving gouty tophi (Sriranganathan et al., 2021). However, as an exogenous protein, it suffers from common drawbacks associated with protein-based drugs, such as susceptibility to hydrolysis, poor stability, and a short half-life. More critically, its high immunogenicity poses risks of allergic reactions and the induction of anti-drug antibodies upon systemic administration, thereby limiting its clinical application (Richette et al., 2017). This scenario underscores the necessity for an effective drug delivery system capable of the simultaneous release of both therapeutic agents (Peng et al., 2023).

In conventional oral therapy, the delivery of agents to the target joint is impeded by hepatic first-pass metabolism and non-specific systemic distribution. This often leads to insufficient local bioavailability within the articular cavity, leading to delayed onset of action, unsatisfactory therapeutic outcomes, and long-term risks of persistent inflammation, recurrent flares (Zhang et al., 2021). In contrast, intra-articular administration has emerged as a promising alternative for gout treatment, offering distinct advantages over systemic and oral routes (Grassot et al., 2025). By directly delivering drugs to the lesion site, intra-articular administration significantly enhances local drug concentration, achieves rapid onset of action, and minimizes systemic drug exposure, thereby reducing the risk of systemic adverse reactions. In recent years, the integration of intra-articular administration with smart responsive microcarriers has become a research hotspot in targeted therapy. These smart microcarriers can respond to specific internal or external stimuli, such as pH, temperature, and ultrasound, to achieve controlled and on-demand drug release, which further improves therapeutic efficacy and reduces side effects compared to traditional intra-articular drug delivery systems (Lin and Hsu, 2025; Wang et al., 2025b).

As a safe exogenous stimulus, ultrasound (US) facilitates efficient drug delivery and targeted therapy in a non-invasive manner. In recent years, its application in the treatment of bone and joint diseases has received increasing attention (Yang et al., 2025). Ultrasound responsive drug delivery systems, including nanocomposites (Liu et al., 2025), hydrogels (Huang et al., 2022; Wang et al., 2025a; Yamakawa et al., 2025), and organic compounds (Hou et al., 2025), have shown promising therapeutic potential in oncology, endocrine disorders, and osteoarthritis (Huang et al., 2025; Lin et al., 2025; Shen et al., 2025; Wang H. et al., 2025). However, their application in the long-term treatment of gouty arthritis remains relatively underexplored.

Intra-articular (IA) administration presents significant challenges for the treatment of joint diseases. The retention of therapeutics at the injection site is often poor; small-molecule drugs are rapidly eliminated via absorption into synovial capillaries and lymphatic drainage (Evans et al., 2014). Consequently, there is a pressing need to design delivery systems with sustained-release properties. Such systems can prolong the duration of action and minimize clearance from the administration site, thereby enhancing local drug retention within the joint cavity.

Hydrogels serve as excellent drug carriers in this context. Their high viscosity promotes drug retention within the joint, facilitating continuous release into surrounding tissues, while their high-water content offers lubricating and soothing effects on inflamed tissues (Pei et al., 2023; Rizzo and Kehr, 2021). Following injection, *in situ* gelation helps maintain joint viscoelasticity (Lin et al., 2025). The hydrogel matrix acts as a reservoir, providing a buffer for slow and sustained drug release. Ultimately, such formulations enhance bioavailability and achieve high local drug concentrations, while simultaneously reducing systemic toxicity by lowering the frequency of administration.

**FIGURE 1:**
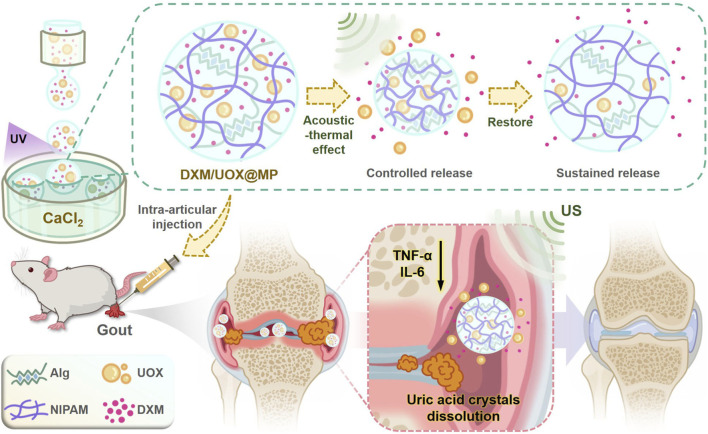
Schematic illustration showed that the DXM/UOX@MPs were fabricated using microfluidic technology and can be responsive to the hyperthermia caused by ultrasound, realizing controlled release of UOX and sustained release of DXM. By intra-articular injecting DXM/UOX@MPs accompanied with ultrasound treatment, uric acid crystals can be dissolved and the inflammation can be suppressed.

The combination of targeted delivery and US stimuli-responsive release enables precise regulation of drug action at the pathological site, providing a new direction for optimizing gout therapy. Based on these, we propose a novel ultrasound-responsive hydrogel microcarrier (DXM/UOX@MPs) constructed via microfluidic electrospray technology. The microcarriers are composed of a double network hydrogel of sodium alginate and thermoresponsive N-isopropylacrylamide (NIPAM), co-loaded with DXM and urate oxidase (UOX). Designed for intra-articular injection in gouty joints, DXM is slowly released to exert long-term anti-inflammatory effects, while high-frequency ultrasound stimulation generates thermal effects that trigger NIPAM network contraction, enabling targeted release of macromolecular UOX to degrade uric acid locally. Notably, our constructed DXM/UOX@MPs exhibit excellent biosafety. In gouty rat models, the combination of DXM/UOX@MPs and ultrasound therapy demonstrated superior therapeutic efficacy: treated rats showed significant reduction in joint swelling and marked resolution of inflammation in the affected joints. As shown in Figure 1, this synergistic therapeutic strategy not only addresses the critical issues of insufficient local drug concentration and lack of targeted release in current gout treatment but also provides a promising therapeutic approach with highly positive clinical application value, especially for patients with refractory gout or poor tolerance to systemic drugs.

## 2 Materials and methods

### 2.1 Fabrication of the DXM/UOX@MPs

First, a capillary with an inner diameter of 300 μm was selected and fixed on a glass slide using resin adhesive, with half of the capillary positioned on the slide and the other half extending beyond the slide. The portion of the capillary outside the slide served as the outlet of the microfluidic channel. A sampling needle was attached to the inlet of the capillary segment on the slide, functioning as the inlet of the microfluidic channel. After verifying the airtightness and patency of the microfluidic chip, it was set aside for subsequent use. Then, a pre-gel solution was prepared, containing 3% (w/v) medium-viscosity sodium alginate, 10% (w/v) NIPAM, and 1% (w/v) photoinitiator 1173. UOX (0.05 U/mL) and DXM (0.1 mg/mL) were then added to this solution, and the mixture was stirred uniformly at room temperature under dark conditions to avoid premature NIPAM cross-linking.

The prepared drug-loaded pre-gel solution was infused into the microfluidic chip via the sampling needle. A 2% (w/v) calcium chloride solution was prepared as the collection medium and placed below the chip outlet. A high-voltage direct current electric field (8 kV) was applied between the chip and the collection solution to induce electrospray. During fabrication, the microspheres in the collection solution were continuously exposed to ultraviolet light to trigger NIPAM cross-linking. The obtained microspheres were rapidly washed three times with phosphate-buffered saline to remove uncrosslinked NIPAM and excess calcium chloride. The cleaned microspheres were then stored in the dark under refrigerated conditions until further use.

### 2.2 Thermal responsiveness of the DXM/UOX@MPs

Based on our previous findings, ultrasonic irradiation at a frequency of 3 MHz, power density of 2 W/cm2 and duty cycle of 50% for 60 s generates sufficient heat to induce NIPAM network contraction without causing damage to normal cells and tissues (Huang et al., 2021). Therefore, these parameters were adopted in the present study to trigger DXM/UOX@MPs shrinkage. Briefly, the prepared DXM/UOX@MPs were placed in a small Petri dish with a diameter of 3.5 cm. The bottom of the dish was coated with ultrasonic coupling gel to ensure efficient acoustic transmission. The ultrasonic probe was then positioned at the bottom of the Petri dish. Stereomicroscopy was performed to record the morphology of microcarriers before and after ultrasonic stimulation. ImageJ software was employed to measure and statistically analyze the diameter of microcarriers.

### 2.3 Drug release kinetics of the DXM/UOX@MPs

To measure UOX release, UOX was conjugated with fluorescein isothiocyanate (FITC) via the reaction between the isothiocyanate group of FITC and the primary amine groups of UOX in a carbonate-bicarbonate buffer (pH 8.5). The FITC-conjugated UOX was then dissolved into the pre-gel solution to fabricate DXM/FITC-UOX@MPs microcarriers following the previously established protocol. The passive release of DXM and UOX from the DXM/FITC-UOX@MPs without ultrasonic stimulation was first measured. The prepared DXM/FITC-UOX@MPs were incubated in phosphate-buffered saline (PBS) at 37 °C. At specific time points, 200 μL of the supernatant from the microcarrier suspension was collected, and an equal volume of fresh PBS was added. The collected samples were analyzed to quantify DXM release via absorbance measurement at 240 nm, and UOX release via fluorescence intensity measurement at an excitation wavelength of 495 nm and an emission wavelength of 519 nm, respectively. For the measurement of ultrasound-triggered release, 60-s ultrasonic stimulation based on the findings of previous research (3 MHz, 2 W/cm2, 50% duty cycle) (Huang et al., 2021)was applied to the microcarriers on Days 0, 1, 2, 3, 4, and 5 before collecting samples. The release ratios of DXM and UOX were determined using the same methods described above for passive release.

### 2.4 *In Vitro* biosafety evaluation of the DXM/UOX@MPs

To verify the biosafety of the hydrogel matrix and the applied drug concentrations, two types of microcarriers were prepared: drug-free microcarriers (MPs) and drug-loaded microcarriers (DXM/UOX@MPs) for subsequent cellular experiments. L929 cells were randomly divided into three experimental groups: the Control group, the MPs + US group, and the DXM/UOX@MPs + US group. No treatment was administered to the Control group. Cells in the MPs + US group were co-cultured with drug-free MPs and subjected to 60 s of ultrasonic stimulation daily. Cells in the DXM/UOX@MPs + US group were co-cultured with DXM/UOX@MPs and received the same daily 60-s ultrasonic stimulation protocol. Cell viability was assessed using the CCK-8 assay at 6 h, 12 h, and 24 h post-seeding. Meanwhile, live-dead cell staining was performed at 24 h, 48 h, and 72 h. The fluorescence signals of stained cells were observed and recorded under an inverted fluorescence microscope.

### 2.5 Evaluation of anti-inflammatory activity of the DXM/UOX@MPs

RAW264.7 macrophage cells were treated with 1 μg/mL lipopolysaccharide (LPS) to induce an inflammatory response. Simultaneously, different experimental interventions, including, co-culture with MPs subjected to 60 s of ultrasonic stimulation one time (MPs + US group), co-culture with DXM (DXM group), co-culture with DXM/UOX@MPs subjected to 60 s of ultrasonic stimulation one time (DXM/UOX@MPs + US group), were applied to the corresponding wells, with blank medium-treated cells serving as the negative control. After 6 and 24 h, the concentrations of pro-inflammatory cytokines, including tumor necrosis factor-α (TNF-α) and interleukin-1β (IL-1β), in the supernatants were quantified using enzyme-linked immunosorbent assay (ELISA) kits following the manufacturers’ protocols. The absorbance values of the reaction solutions were measured at 450 nm using a microplate reader, and the cytokine concentrations were calculated based on the standard curves established with known concentrations of recombinant TNF-α and IL-1β.

### 2.6 Animal experiments

All animal experiments adhered to the guidelines set by the Animal Ethics Committee of Drum Tower Hospital affiliated to the Medical School of Nanjing University (No. 2023AE02011). Gout was induced by intra-articular injection of 100 μL sterile monosodium urate (MSU) crystal suspension (60 mg/mL) into the right ankle joint of rats, and model success was verified by the presence of joint swelling, redness and restricted mobility at 24 h post-injection. Rats with successfully established gout models were randomly divided into 6 groups: Control, MPs + US, DXM, UOX, DXM/UOX@MPs, and DXM/UOX@MPs + US. We used n = 6 rats per group for all *in vivo* experiments, Photographic records of the hind paws of rats in each group were taken on Days 1, 3, and 7 after different treatments. On Day 7, the ankle temperatures of rats in each group were measured and recorded using an infrared thermal imager. After 7 days, the experimental ankle joints were harvested for immunohistochemical staining and subsequent evaluation.

### 2.7 Statistical analysis

All statistical computations were carried out with the aid of Origin 2019b software, with analyses implemented for applicable datasets. Experimental data are expressed as the mean ± standard deviation. Intergroup comparisons were executed utilizing a one-way ANOVA followed by Tukey’s post-hoc test. A p-value less than 0.05 was defined as indicative of statistically significant differences.

## 3 Results

**FIGURE 2:**
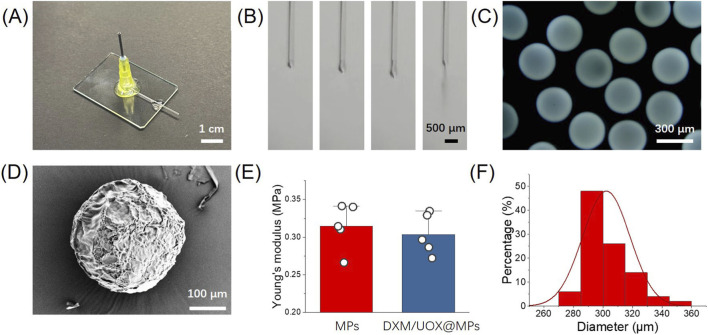
Fabrication of the DXM/UOX@MPs. **(A)** Photograph of a microfluidic chip used in this study. **(B)** High-speed camera images of the droplet formation and falling at the microfluidic chip outlet. **(C)** Stereomicroscope image of the obtained DXM/UOX@MPs. **(D)** SEM image of a DXM/UOX@MP. **(E)** Young’s modulus of microparticles (MPs) and DXM/UOX@MPs (n = 5). **(F)** Diameter distribution of the DXM/UOX@MPs (n = 50).

We first constructed the DXM/UOX@MPs microcarriers using microfluidic electrospray technology. The DXM and UOX co-loaded sodium alginate (ALG)/NIPAM solution was used as the dispersed phase, which formed droplets at the microfluidic chip outlet and dropped into a CaCl_2_ collection solution. Here, alginate cross-linked with Ca^2+^ to form solid calcium alginate, while continuous ultraviolet irradiation of the collection solution induced NIPAM cross-linking, resulting in the final ALG/NIPAM double-network hydrogel microcarriers. Figure 2A shows a photograph of the hand-assembled microfluidic chip, composed of capillaries, a glass slide, and a sampling needle. Figure 2B depicts rapid droplet formation and falling at the microfluidic chip outlet, driven by high-voltage direct current. The polarized solution formed a Taylor cone at the outlet, which broke into droplets. Figure 2C reveals that the obtained microcarriers are uniform, monodisperse spherical particles. Figure 2D provides a scanning electron microscopy (SEM) image of a single microcarrier, showing its surface morphology. Meanwhile, we compared the Young’s modulus of drug-free and drug-loaded hydrogel microspheres. The results demonstrated no significant difference between the two groups, indicating that the encapsulation of the two drugs did not compromise the elastic properties of the hydrogel microcarriers (Figure 2E). Ultimately, the DXM/UOX@MPs fabricated in this study exhibited a size of 302.28 ± 15.93 μm, with excellent uniformity that ensures their injectability (Figure 2F).

**FIGURE 3:**
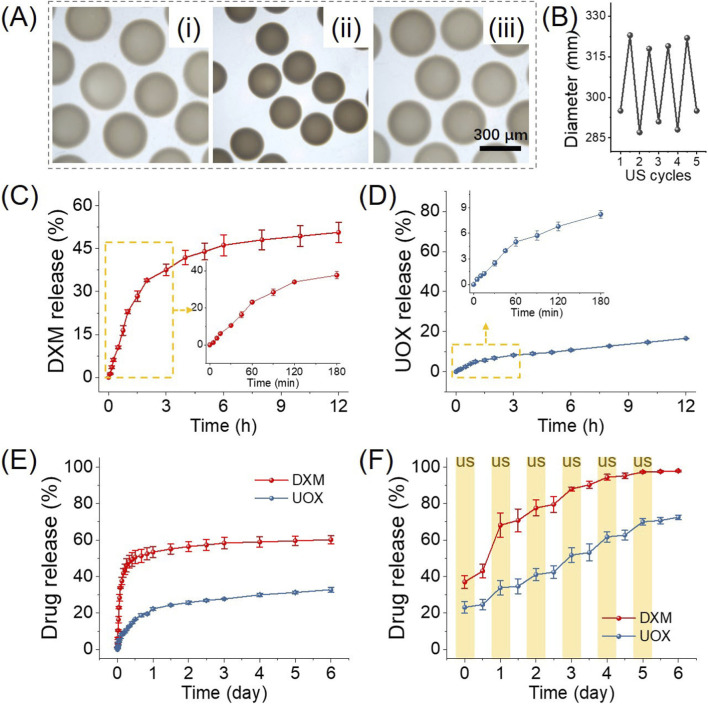
Thermoresponsiveness and drug release kinetics of the DXM/UOX@MPs. **(A)** Stereomicroscopic images of the DXM/UOX@MPs before (i), during (ii), and after (iii) ultrasonic heating. **(B)** Microcarriers’ diameter variation during 5 ultrasonic stimulation cycles. **(C,D)** The DXM and UOX release in 12 h. **(E)** The long-term DXM and UOX release for 6 days **(F)** The long-term DXM and UOX release with daily 60-s ultrasonic stimulation. All release experiments were performed in triplicate.

Following the successful fabrication of the DXM/UOX@MPs, a systematic characterization was conducted to evaluate their thermoresponsive behavior and drug release performance. This characterization aimed to validate whether the microcarriers could exhibit the designed stimulus-responsive properties and achieve controlled payload delivery, laying a foundational basis for subsequent *in vitro* and *in vivo* evaluations. Figure 3A presents the contraction and recovery process of DXM/UOX@MPs during ultrasonic heating. Initially, the DXM/UOX@MPs exhibited a well-defined, monodisperse spherical morphology, consistent with the structural uniformity expected from the microfluidic electrospray fabrication process. Upon exposure to ultrasonic stimulation for 60 s, the microspheres underwent widespread, homogeneous contraction. This macroscopic volume reduction arose from the collapse of the thermoresponsive NIPAM network, which was triggered by the localized heat generated by the ultrasonic energy. When the ultrasonic stimulus was discontinued, the NIPAM network gradually re-swelled as the system cooled to ambient temperature, allowing the microspheres to revert progressively to their original spherical shape and size. This reversible shrinkage-recovery cycle directly demonstrates the microcarriers’ ability to respond dynamically to thermal cues from external stimulation. Consistently, Figure 3B depicts the diameter variation of microcarriers over 5 ultrasonic heating cycles. The periodic fluctuations in particle size indicate that the microcarriers exhibit robust thermoresponsivity and excellent repeatability of the volume change behavior.

Subsequently, we determined the release of DXM and UOX and compared the differences in drug release under conditions with and without ultrasonic stimulation. As illustrated in Figure 3C, within 3 h, 37.57% ± 1.99% of DXM was released, followed by a gradual plateau, confirming sustained DXM release via the microcarrier matrix. In contrast, only 16.60% ± 0.12% of UOX was released within 12 h, with minimal further release, indicating UOX was tightly retained in the microcarriers under static conditions (Figure 3D). Then, we measured the long-term passive release of both agents. DXM exhibited faster release, which reached 53.46% ± 3.11% by 1 day, while UOX showed slow release, which showed 32.84% ± 1.26% release by 6 days, demonstrating the microcarriers’ ability to enable prolonged, differential passive release of the two drugs (Figure 3E). Notably, ultrasound stimulation can significantly enhance the release of both agents. As shown in Figure 3F, upon daily US stimulation (yellow-shaded intervals), both DXM and UOX release accelerated sharply relative to passive release. DXM reached 97.95% ± 0.38% cumulative release by 6 days, while UOX reached 72.47% ± 1.28%, both significantly higher and faster than their passive counterparts.

**FIGURE 4:**
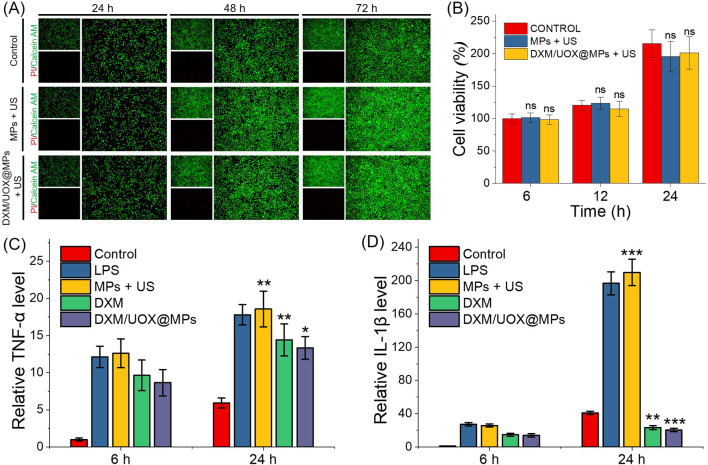
Biocompatibility evaluation of the DXM/UOX@MPs and ultrasonic stimulation. **(A)** live-dead staining results of L929 cells at 24 h, 48 h, and 72 h **(B)** CCK-8 assay for the three groups at 6 h, 12 h, and 24 h **(C,D)** The TNF-α and IL-1β expressions in RAW264.7 cells after different treatment. Data are expressed as mean ± standard deviation (n = 3). *p < 0.05, **p < 0.01, ***p < 0.001 indicate statistically significant differences.

After successfully validating the ultrasonic responsiveness and controllable drug release capacity of the DXM/UOX@MPs microcarriers, we proceeded to assess their biosafety, a critical prerequisite for any biomedical material intended for *in vivo* applications. Figure 4A presents live-dead staining results of L929 cells across three experimental groups (Control, MPs + US, and DXM/UOX@MPs + US) at 24 h, 48 h, and 72 h. In all groups, the cells exhibited predominantly green fluorescence with minimal red fluorescence over the 72-h period, suggesting that exposure to the microcarriers, ultrasonic stimulation, or the DXM/UOX@MPs formulation did not induce significant cell death. Moreover, Figure 4B shows the cell viability data quantified via CCK-8 assay for the three groups at 6 h, 12 h, and 24 h. All groups maintained high cell viability at each time point, and no statistically significant differences were observed between groups. These results collectively demonstrate the favorable biosafety of the microcarriers, their loaded components, and the ultrasonic stimulation protocol.

Next, we evaluated the anti-inflammatory performance of the DXM-loaded microcarriers by assessing tumor necrosis factor-α (TNF-α) and interleukin-1β (IL-1β) expression in RAW264.7 macrophages, as shown in Figures 4C,D. At 6 h, lipopolysaccharide (LPS) stimulation significantly elevated TNF-α levels relative to the control group, confirming successful inflammatory activation. In contrast, the MPs + US, DXM, and DXM/UOX@MPs groups exhibited markedly lower TNF-α levels than the LPS group. At 24 h, TNF-α levels remained high in the LPS group, and the MPs + US group also maintained elevated levels. The DXM and DXM/UOX@MPs groups displayed further reductions in TNF-α, with the DXM/UOX@MPs group showing the most pronounced suppression, resulting in TNF-α levels significantly lower than those of the LPS group. On the other hand, at 6 h, LPS stimulation induced a significant increase in IL-1β levels, while the MPs + US, DXM, and DXM/UOX@MPs groups all had significantly reduced IL-1β levels relative to the LPS group. The DXM/UOX@MPs group exhibited IL-1β levels closest to the control. At 24 h, IL-1β levels in the LPS group increased sharply, whereas the MPs + US group showed a moderate elevation. The DXM and DXM/UOX@MPs groups displayed substantial reductions in IL-1β, with the DXM/UOX@MPs group showing far lower IL-1β levels than the DXM group and significantly reduced levels compared to the LPS group.

**FIGURE 5:**
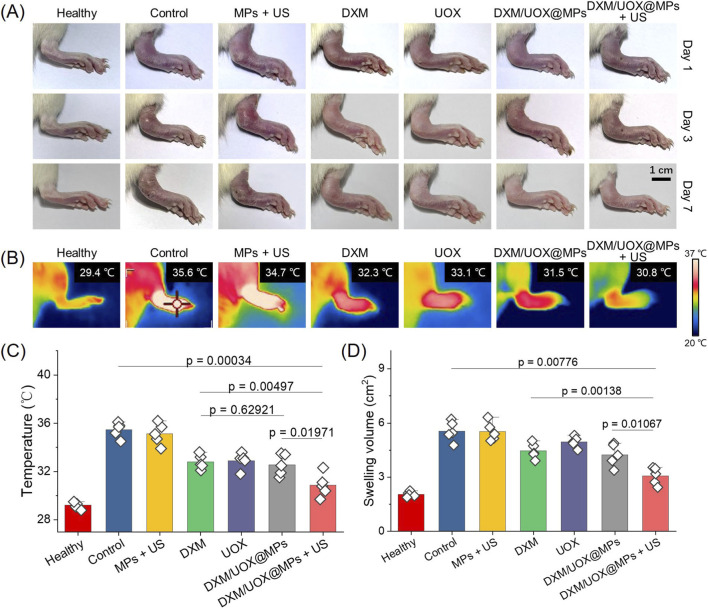
DXM/UOX@MPs treating gouty rat models. **(A)** Digital photos of the hind paws of gouty rats from different groups on day 1, 3, and 7. **(B)** Thermal images of the hind paws of gouty rats from different groups on day 7. **(C,D)** Statistical analyses of the paw temperature and swelling volume of the hind paws of gouty rats from different groups on day 7.

Following the validation of biosafety for DXM/UOX@MPs and ultrasonic stimulation, therapeutic evaluations were conducted in a gout rat model. Rats with successfully induced gout were randomly assigned to 6 groups: Control, MPs + US, DXM, UOX, DXM/UOX@MPs, and DXM/UOX@MPs + US. The Control group received no intervention. The MPs + US group was treated with intra-articular injection of drug-free microspheres plus daily 60-s ultrasonic stimulation. The DXM and UOX group received intra-articular injection of DXM and UOX solution, respectively. The DXM/UOX@MPs group received intra-articular injection of DXM/UOX@MPs without ultrasonic stimulation. The DXM/UOX@MPs + US group received both intra-articular DXM/UOX@MPs injection and daily 60-s ultrasonic stimulation. Photographic records of rat ankle joints on Days 1, 3, and 7 showed consistent severe swelling in the Control and MPs + US groups, with no observable differences between these two groups (Figure 5A). The DXM/UOX@MPs group exhibited milder swelling, while the DXM/UOX@MPs + US group showed the most significant reduction in swelling, with joint morphology approaching that of the healthy control. On Day 7, thermal imaging revealed markedly elevated temperatures in the Control and MPs + US groups, whereas temperatures in the DXM/UOX@MPs and DXM/UOX@MPs + US groups were significantly lower; the DXM/UOX@MPs + US group displayed the most prominent temperature reduction, close to the healthy baseline (Figure 5B).

As illustrated in Figures 5C,D, quantitative analyses of ankle temperature and swelling volume on Day 7 confirmed these observations. The Control and MPs + US groups had comparable high temperatures and large swelling volumes. The DXM, UOX, and DXM/UOX@MPs group showed modest decreases in both parameters, while the DXM/UOX@MPs + US group exhibited statistically significant reductions in temperature and swelling volume, with values significantly lower than those of the DXM/UOX@MPs group. These results collectively indicate that DXM/UOX@MPs combined with ultrasonic stimulation exerts therapeutic effects on gout in rats.

**FIGURE 6:**
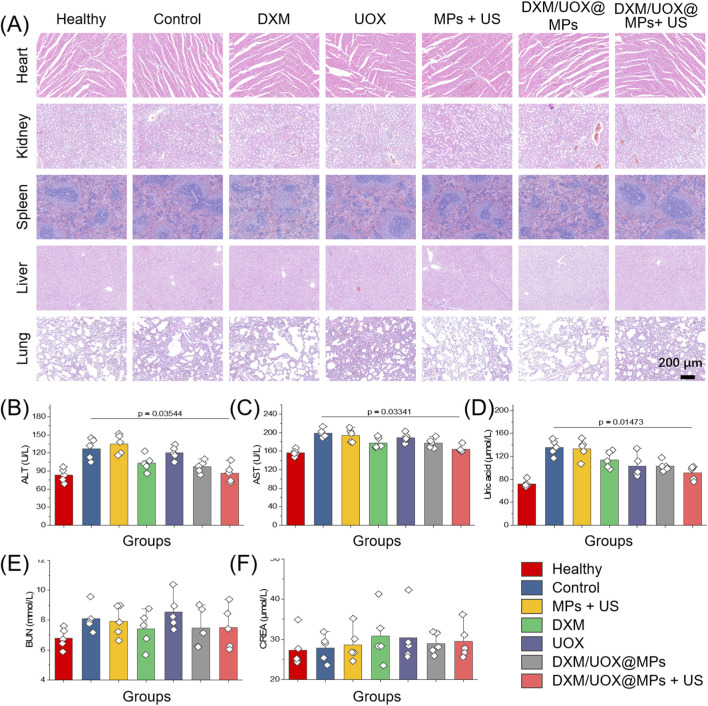
Biosafety evaluation and biochemical indicators of rats. **(A)** H&E staining images of the main organs of rats from different groups. **(B–F)** Hepatic and renal function tests of GA rats after administration of drugs. ALT, alaninetransaminase; AST, aspartate transaminase; BUN, blood urea nitrogen; CREA, creatinine.

Hematoxylin and eosin (H&E) staining of major organs across all groups revealed that tissue architecture remained indistinguishable from the Healthy group, with no signs of necrosis, inflammation, or structural damage (Figure 6A). This histological evidence was also supported by serum biochemical analysis (Figures 6B–F), where the Control group exhibited elevated levels of ALT, AST, and urea, indicating subclinical organ stress from untreated gout, while all treatment groups, including DXM/UOX@MPs + US, maintained biochemical marker levels comparable to the Healthy group, with no statistically significant differences. Notably, the DXM/UOX@MPs + US group showed the most pronounced normalization of urea levels, reflecting improved uric acid clearance. These findings collectively confirm the systemic biosafety of the DXM/UOX@MPs therapy, demonstrating that the treatment does not induce off-target toxicity even with repeated ultrasonic stimulation, while also providing therapeutic benefit by reducing systemic metabolic stress. This validates the biocompatibility of the microcarrier system and its safety for *in vivo* application, reinforcing its potential as a safe and effective treatment for gout.

**FIGURE 7:**
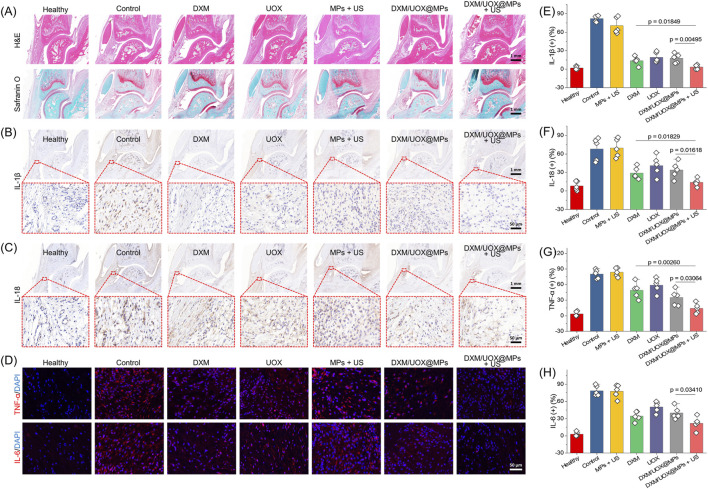
Histological and immunohistochemical analysis. **(A)** Representative H&E staining (top) and Safranin O staining (bottom) of rat ankle joint sections. **(B)** Immunohistochemical staining of IL-1β and **(C)** IL-18, with insets displaying high-magnification views of the synovial inflammatory infiltration area. **(D)** Immunofluorescence staining of TNF-α and IL-6. **(E–H)** Quantitative analysis of pro-inflammatory cytokines in joint tissues. The bar graphs show the percentage of positive (+) expression for **(E)** IL-1β, **(F)** IL-18, **(G)** TNF-α, and **(H)** IL-6 across the different experimental groups: Healthy, Control, MPs + US, DXM, UOX, DXM/UOX@MPs, and DXM/UOX@MPs + US. Data are expressed as mean ± SD (n = 5 per group). Specific p-values for key comparisons are labeled above the corresponding brackets.

Histopathological evaluation by H&E and Safranin O staining revealed that the Control and MPs + US group exhibited severe synovial hyperplasia, dense inflammatory cell infiltration, and progressive cartilage degradation (Figure 7A). In contrast, other treatment groups showed improved joint morphology, with the DXM/UOX@MPs + US group exhibiting the most pronounced structural preservation, characterized with minimal synovial thickening, near-normal synovial architecture, and robust Safranin O retention in articular cartilage, which is consistent with sustained extracellular matrix integrity. Moreover, immunohistochemical analysis of IL-1β and IL-18 expression demonstrated strong, diffuse positive signals in the synovial lining and perivascular inflammatory foci of the Control group and the MPs + US group (Figures 7B,C). Treatment significantly attenuated cytokine immunoreactivity in a group-dependent manner. Notably, the DXM/UOX@MPs + US group exhibited the greatest suppression, characterized by reduced IL-1β and IL-18 staining intensities to levels comparable to those observed in the Healthy group. This suggests potent, synergistic inhibition of inflammasome-dependent cytokine maturation and release. Immunofluorescence quantification further revealed intense cytoplasmic TNF-α and IL-6 signals in infiltrating immune cells of the Control group (Figure 7D). Using ELISA to quantify joint homogenates and semi-quantitative immunohistochemistry/immunofluorescence analyses, we obtained numerical measurements of IL-1β, IL-18, TNF-α, and IL-6 levels in each group. Gout/PBS group showed significantly elevated levels of all cytokines compared to healthy controls (p < 0.001). Free drug treatment moderately reduced cytokine levels (p < 0.05 vs. gout group). DXM/UOX@MPs + US group demonstrated the most significant reduction in all cytokines, especially IL-1β (p < 0.01 vs. other groups). These quantitative data have been showed in (Figures 7E–H).

The therapeutic interventions including DXM/UOX@MPs application reduced fluorescence intensity relative to control, with the DXM/UOX@MPs + US group showing the lowest integrated density for both cytokines, corroborating its superior efficacy in suppressing the pro-inflammatory cytokine cascade. Collectively, these multimodal histological and molecular analyses demonstrate that DXM/UOX@MPs + US exerts comprehensive anti-inflammatory effects in gouty arthritis, effectively mitigating synovitis and preserving cartilage structure.

## 4 Discussion

The characterization results confirm that the microfluidic electrospray strategy successfully fabricates DXM/UOX@MPs with desired structural properties. First, the double-network cross-linking process, combining rapid Ca^2+^-mediated alginate gelation and ultraviolet-triggered NIPAM polymerization, ensures the microcarriers maintain structural integrity in physiological environments, which is critical for controlled drug release. The hand-assembled microfluidic chip is a cost-effective and flexible setup. Although its manual construction may introduce minor variability, the consistent droplet formation indicates the chip’s reliability for producing microcarriers at lab scale. The electrospray mechanism is key to the microcarriers’ uniformity. The high-voltage polarization generates stable Taylor cones, minimizing droplet size variation, which is reflected in the monodisperse, spherical particles. Specifically, this homogeneity is essential for reproducible drug loading and predictable release behavior across batches. The SEM image further verifies the microcarriers’ well-defined spherical structure and porous surface, which can facilitate drug diffusion and ultrasound-responsive deformation, supporting the subsequent therapeutic function of the microcarriers.

Next, the contraction-recovery behavior of DXM/UOX@MPs directly reflects the thermoresponsive property of the NIPAM network. ultrasonic heat triggers NIPAM chain collapse, which translates to macroscopic microcarrier shrinkage, and the network re-swells upon cooling. This reversible volume change is the basis for stimulus-responsive drug release. The consistent diameter fluctuations over 5 cycles highlight the structural stability of the ALG/NIPAM double network, as the physical cross-linking of alginate provides mechanical support, while the NIPAM network maintains its thermoresponsive functionality, ensuring repeatable responsiveness without structural degradation. The enhanced DXM and UOX release under ultrasonic heating is attributed to the contraction-induced network compression. The shrunken NIPAM chains squeeze the hydrogel matrix, facilitating the diffusion of DXM and UOX molecules. This stimulus-dependent release profile is critical for gout therapy, as it enables on-demand UOX delivery to the joint cavity upon ultrasonic stimulation, while minimizing unintended drug leakage under physiological conditions.

We observed that the release profiles of DXM and UOX are different, which can be attributed to the distinct physicochemical properties of the two agents. DXM, as a small hydrophobic molecule (MW = 392.5 Da), exhibits faster diffusion from the collapsed PNIPAM network upon ultrasound-triggered phase transition. Its hydrophobic nature also promotes interaction with the hydrophobic domains of collapsed PNIPAM, leading to a burst release pattern. UOX, as a large hydrophilic protein (MW ∼ 34 kDa), experiences greater steric hindrance within the hydrogel matrix. Its release is primarily governed by the pore size changes of the PNIPAM network rather than simple diffusion, resulting in a slower, more sustained release profile. Additionally, the different loading mechanisms (hydrophobic interaction for DXM vs. physical entrapment for UOX) contribute to the distinct release kinetics. We have emphasized that this differential release is advantageous for achieving immediate anti-inflammatory effects (DXM) followed by sustained uric acid depletion (UOX). Together, these results validate that the microcarriers integrate effective thermoresponsivity and controllable drug release, which are key functional attributes for targeted gout treatment.

To confirm that the microcarriers, along with their associated ultrasonic stimulation protocol, do not induce cytotoxicity or adverse cellular responses, ensuring their compatibility with biological environments before advancing to preclinical *in vivo* evaluations, we evaluated the biocompatibility using L929 cells. The live-dead staining and CCK-8 viability data confirm the excellent biosafety profile of the DXM/UOX@MPs system, as well as the compatibility of ultrasonic stimulation with cellular viability. The consistent prevalence of viable cells across all groups indicates that neither the ALG/NIPAM hydrogel matrix, the loaded DXM and UOX payloads, nor the ultrasonic heating process elicits cytotoxic effects on L929 cells. The lack of significant differences in cell viability between groups further supports that the microcarriers and associated stimulation do not compromise cellular function. This biosafety is critical for the translational potential of the system, since joint cavity injection requires materials and treatments that avoid local tissue damage, and these results validate that the DXM/UOX@MPs + US strategy meets this prerequisite. Additionally, the sustained high viability over time suggests the system can persist in the joint microenvironment without inducing adverse cellular responses, laying a foundation for subsequent *in vivo* safety and efficacy evaluations.

The TNF-α and IL-1β expression profiles confirm that DXM/UOX@MPs effectively suppresses LPS-induced inflammatory responses in RAW264.7 macrophages, with superior performance to standalone MPs + US or DXM treatments. At 6 h, all experimental groups attenuated initial cytokine release, but DXM/UOX@MPs exhibited the strongest early inhibition of IL-1β, likely due to the sustained release of DXM from the microcarrier matrix. By 24 h, the prolonged anti-inflammatory effect of DXM/UOX@MPs became more evident: it maintained robust suppression of both TNF-α and IL-1β, whereas the MPs + US group showed diminished efficacy, and the DXM group had less potent inhibition than the microcarrier formulation. This enhanced performance of DXM/UOX@MPs may stem from the synergistic effects of sustained DXM release (for continuous anti-inflammatory action) and the microcarrier’s structural properties (which may prolong local drug retention). The significant reduction in key pro-inflammatory cytokines (TNF-α and IL-1β)—central mediators of gout-associated joint inflammation, which validates that DXM/UOX@MPs targets the core inflammatory pathway of gout, supporting its potential as an anti-inflammatory agent for gout therapy.

Having established the favorable *in vitro* biosafety of the DXM/UOX@MPs microcarriers and their compatible ultrasonic stimulation protocol in cellular models, we next evaluated the therapeutic efficacy of this system in a preclinical gout rat model to translate these foundational safety findings into functional performance. The *in vivo* therapeutic outcomes demonstrate that the DXM/UOX@MPs + US strategy effectively alleviates gout-related joint swelling and inflammation, while other groups show limited or no efficacy. The lack of improvement in the Control and MPs + US groups confirms that neither no intervention nor drug-free microspheres with ultrasound elicit therapeutic effects, ruling out non-specific responses to the microcarrier or stimulation. The modest efficacy of the DXM/UOX@MPs group aligns with the passive, slow release of DXM. This provides partial anti-inflammatory effects but cannot achieve targeted UOX release, limiting its ability to clear local uric acid. In contrast, the superior performance of the DXM/UOX@MPs + US group stems from the synergistic action of sustained DXM release and ultrasound-triggered UOX release. The ultrasonic stimulation not only drives UOX release but also may enhance local drug diffusion, further amplifying therapeutic effects. The reductions in joint temperature and swelling volume directly reflect the attenuation of the gout-related immune response, consistent with the dual mechanisms of anti-inflammation and uric acid clearance. In addition, for arthritis-induced joint damage, histopathological analysis revealed that the combination of DXM/UOX-loaded microbubbles with ultrasound (DXM/UOX@MPs + US) significantly alleviated synovial inflammation, reduced bone erosion, and preserved cartilage integrity compared to the model control group. Notably, this treatment prevented severe loss of proteoglycans in the cartilage matrix and reduced macrophage-rich inflammatory infiltrates. While individual variability in response was observed, the DXM/UOX@MPs + US group demonstrated the most pronounced chondroprotective and anti-inflammatory effects among all treatment groups, highlighting the potential of ultrasound-targeted microbubble destruction as a strategy for enhanced drug delivery in arthritis therapy. These results support that the stimulus-responsive DXM/UOX@MPs system, when paired with ultrasonic activation, is a promising targeted therapy for gout, addressing both inflammatory and metabolic components of the disease.

## 5 Conclusion

In summary, this study reports the rational design and functional validation of an ultrasound-responsive microcarrier platform (DXM/UOX@MPs) for sequential, on-demand co-delivery of dexamethasone (DXM) and uricase (UOX) in gouty arthritis. The system exhibits a dual-phase therapeutic mechanism, including initial passive diffusion of DXM enables rapid suppression of acute synovial inflammation, whereas sustained enzymatic release of UOX promotes prolonged uric acid degradation and metabolic normalization. Critically, ultrasound stimulation triggered accelerated and spatially localized release of both payloads, enhancing release kinetics, improving intra-articular bioavailability, and minimizing off-target exposure. Comprehensive biocompatibility and safety assessments and serum biochemical profiling such as ALT, AST, BUN, and CRE, confirmed the absence of systemic toxicity. In an MSU-induced rat model of gouty arthritis, DXM/UOX@MPs + US treatment significantly attenuated NLRP3 inflammasome activation in synovial tissue, markedly reduced expression and secretion of key pro-inflammatory cytokines including IL-1β, IL-18, TNF-α, and IL-6, alleviated synovial hyperplasia and inflammatory infiltration, and preserved cartilage proteoglycan content and structural integrity. Collectively, these data establish DXM/UOX@MPs + US as a safe, spatiotemporally controllable, and therapeutically synergistic strategy that simultaneously targets the immunological drivers and metabolic etiology of gouty arthritis, thereby addressing both acute flares and chronic joint damage.

## Data Availability

The original contributions presented in the study are included in the article, further inquiries can be directed to the corresponding authors.
